# Differential cytotoxic effects of graphene and graphene oxide on skin keratinocytes

**DOI:** 10.1038/srep40572

**Published:** 2017-01-12

**Authors:** Marco Pelin, Laura Fusco, Verónica León, Cristina Martín, Alejandro Criado, Silvio Sosa, Ester Vázquez, Aurelia Tubaro, Maurizio Prato

**Affiliations:** 1 Department of Life Sciences, University of Trieste, 34127 Trieste, Italy; 2 Department of Chemical and Pharmaceutical Sciences, University of Trieste, 34127 Trieste, Italy; 3 Department of Organic Chemistry, Facultad de Ciencias y Tecnologías Químicas-IRICA, University of Castilla-La Mancha, 13071 Ciudad Real, Spain; 4 CIC BiomaGUNE, Parque Tecnológico de San Sebastián, Paseo Miramón, 182, 20009 San Sebastián (Guipúzcoa), Spain; 5 Basque Foundation for Science, Ikerbasque, Bilbao 48013, Spain

## Abstract

Impressive properties make graphene-based materials (GBMs) promising tools for nanoelectronics and biomedicine. However, safety concerns need to be cleared before mass production of GBMs starts. As skin, together with lungs, displays the highest exposure to GBMs, it is of fundamental importance to understand what happens when GBMs get in contact with skin cells. The present study was carried out on HaCaT keratinocytes, an *in vitro* model of skin toxicity, on which the effects of four GBMs were evaluated: a few layer graphene, prepared by ball-milling treatment (FLG), and three samples of graphene oxide (GOs, a research-grade GO1, and two commercial GOs, GO2 and GO3). Even though no significant effects were observed after 24 h, after 72 h the less oxidized compound (FLG) was the less cytotoxic, inducing mitochondrial and plasma-membrane damages with EC_50_s of 62.8 μg/mL (WST-8 assay) and 45.5 μg/mL (propidium iodide uptake), respectively. By contrast, the largest and most oxidized compound, GO3, was the most cytotoxic, inducing mitochondrial and plasma-membrane damages with EC_50_s of 5.4 and 2.9 μg/mL, respectively. These results suggest that only high concentrations and long exposure times to FLG and GOs could impair mitochondrial activity associated with plasma membrane damage, suggesting low cytotoxic effects at the skin level.

Graphene consists of a single atom thick, two-dimensional sheet of sp^2^–carbons forming six-membered rings in a honeycomb structure, produced, for the first time, by scotch tape exfoliation of graphite^1^,^2^,^3^,^4^. Graphene oxide (GO), a highly oxidized form of chemically modified graphene^5^,^6^,^7^,^8^,^9^,^10^, can be obtained by oxidation of graphite under strongly acidic conditions^11^,^12^. The structure of GO consists of a basal plane and edges decorated with oxygen-containing functional groups, such as hydroxyl, epoxy and carbonyl groups^13^,^14^.

In recent years, graphene has drawn tremendous attention due to its unique physicochemical properties, including high surface-to-volume ratio, strong mechanical strength, remarkable optical transmittance as well as extraordinary electrical and thermal conductivity^15^,^16^,^17^,^18^,^19^,^20^,^21^,^22^.

New functionalization methods of graphene nanocomposites or hybrids can be applied to obtain other graphene-based materials (GBMs) with suitable properties to improve and expand their potential range of novel applications^23^,^24^,^25^,^26^,^27^,^28^,^29^,^30^,^31^,^32^. However, despite the huge interest in GBM technological progress, the potential human risk related to these compounds does not seem to be completely clarified^33^,^34^,^35^. Humans can be exposed to GBMs by different routes, especially by inhalation, skin, and oral exposures, or even direct injection through biomedical interventions^36^. Among them, however, cutaneous and inhalational exposures are the most viable exposure routes during the production of GBMs, especially as dry powders by thermal exfoliation of graphite as well as during their use and disposal. Skin is the largest organ of the human body and, despite its barrier properties, represents one of the main and largest surfaces through which nanomaterials can enter into the body^37^. Nevertheless, in contrast to the inhalational exposure, toxic effects of GBMs in humans after cutaneous exposure remain largely unexplored, despite cutaneous contact to graphite and other carbon nanomaterials have been associated with increased incidence of skin diseases, such as airborne irritant contact dermatitis, hyperkeratosis and naevi^38^,^39^.

Hence, considering the potential cutaneous effect of these carbon nanomaterials, we evaluated the *in vitro* effects of GBMs on human skin HaCaT keratinocytes, a spontaneously immortalized non-tumor cell line, widely used as a first-round screening to evaluate the toxicity of several compounds at the skin level^40^. Indeed, this *in vitro* model was recently used to investigate also the biocompatibility against normal skin cells of GBMs as anti-cancer therapy^41^. To this purpose, we selected and characterized four different materials: two research grade materials (few layer graphene, FLG, and graphene oxide 1, GO1) and two commercial GOs, prepared using two different starting materials (carbon nanofibers, GO2 and graphite, GO3) with notable differences in the amount of defects and oxygen content.

## Results and Discussion

### Characterization of FLG and GO

A sample of FLG was prepared by ball-milling treatment, according to published procedures^42^,^43^,^44^. A sample of GO (GO1) was prepared by the common Hummer’s method. Two commercially available GOs, GO2 and GO3, were used as received. The four GBMs were thoroughly characterized to determine the influence on the toxicity of the lateral dimension and chemical composition (Table 1). In particular, the graphene derivatives were characterized using techniques such as transmission electron microscopy (TEM), elemental analysis (EA), thermogravimetric analysis (TGA), X-ray photoelectron spectroscopy (XPS) and Raman spectroscopy.

**Figure 1:**
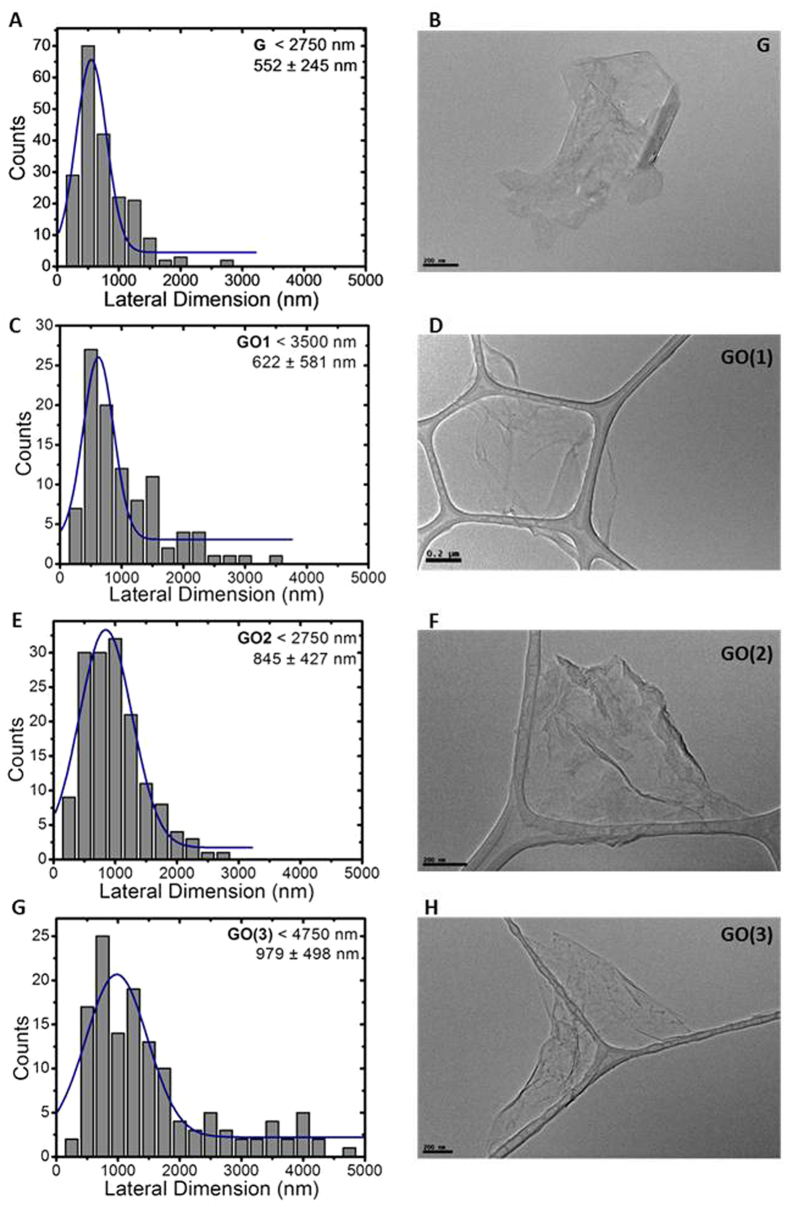
Lateral dimension distribution from TEM images (**A,C**,**E**,**G**) and representative TEM images (**B**,**D**,**F**,**H**) of GBMs in culture medium. Scale bar 200 nm.

Lateral dimension distribution analysis using High-Resolution Transmission Electron Microscopy (HRTEM) of the corresponding GBMs in culture medium (Fig. 1) showed that FLG and GO1 possess the smallest lateral sizes, with average lateral dimensions of 552 and 622 nm, respectively. GO2 and GO3 exhibited a higher size, corresponding roughly to double lateral dimensions compared to FLG.

By elemental analysis and XPS spectroscopy, the chemical composition, C/O ratio and type of oxygen-containing functional groups were determined (Table 1). Elemental analysis gave average values of oxygen compatible with the weight loss at 700 °C observed by TGA [Supporting Information, Figure S1A], also in accordance with the C/O atomic ratio obtained from XPS analysis. As expected, the mechano-chemically exfoliated graphene derivative, FLG, did not show a significant amount of oxygen content, whereas GO1, GO2 and GO3 exhibited a large and similar content of oxygen groups, consisting mainly of epoxy and hydroxyl groups, as deduced by high resolution XPS spectroscopy (Supporting Information, Figure S2). In addition, the GO samples present small quantities of sulfur in the chemical composition, especially important in the case of GO1, with about 10%, mainly as sulfoxide groups^45^,^46^.

Raman spectroscopy can be efficiently used to monitor the quality of graphene layers among other structural characteristics^47^. The differences between the Raman spectra of FLG and the GO derivatives make it clear the contrast between these materials (Supporting Information, Figure S1B). For FLG, the D to G band intensity ratio was calculated at different locations, giving a significantly low value (0.2 ± 0.05), corroborating the low number of defects generated by the ball milling treatment in comparison with the strong oxidation conditions used in the preparation of GO derivatives. GO1, GO2 and GO3 exhibit broad D and G Raman bands and in addition, with a bump instead of the usual 2D band common to graphene structures.

### Effects of FLG and GOs on cell viability

The different physicochemical properties of GBMs, their composition, shape, size and starting material used for their production could ultimately influence their interaction with cells and their cytotoxicity^37^,^48^. Their effects were investigated on human HaCaT skin keratinocytes, a spontaneously immortalized non-tumor cell line as an *in vitro* model for first screening of cutaneous toxicity^40^.

**Figure 2:**
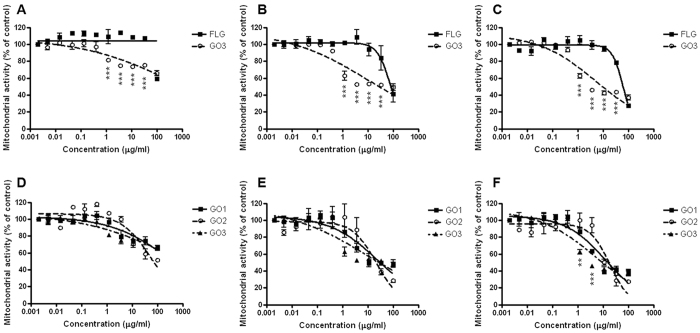
Effect of FLG and GOs on HaCaT cells mitochondrial activity evaluated by the WST-8 assay. Comparison between the less (FLG) and most (GO3) oxidized GBMs after 24 h (**A**), 48 h (**B**) and 72 h (**C**) exposure. Comparison between similarly oxidized GBMs differing by average lateral dimension (GO1, GO2, GO3) after 24 h (**D**), 48 h (**E**) and 72 h (**F**) exposure. Data are the mean ± SE of 3 independent experiments performed in triplicate. Statistical differences: **p < 0.01; ***p < 0.001 (Two-way ANOVA and Bonferroni’s post test).

Initially, the effects of FLG and GOs on cell viability were evaluated by means of mitochondrial activity of HaCaT cells after different exposure times (24 up to 72 h) by the WST-8 assay (Fig. 2). This assay, widely used to investigate mitochondrial damages of different GBMs on a wide range of cell models^49^,^50^,^51^, was preferred to the MTT assay since the latter can generate a nonspecific signal due to a possible spontaneous reduction of the MTT reagent by GBMs, leading to false positive overestimation of cell viability^52^.

Figure 2 (panels A–C) shows a comparison between the less (FLG) and most oxidized (GO3) GBMs after 24 (panel A), 48 (panel B) and 72 hours of exposure (panel C). While the 24 h exposure to FLG (0.005–100 μg/mL) did not exert any effect, a significant reduction of mitochondrial activity was observed after 48 h of exposure at the concentration equal or higher than 30 μg/mL (16% of mitochondrial activity reduction). On the other hand, GO3 exerts significant effects already after 24 h exposure, inducing a significant reduction of the mitochondrial activity starting at the concentrations of 3 μg/mL (25% reduction). However, the relatively low cytotoxic effects observed after 24 and 48 h did not allow the computation of EC_50_ values, which were calculated only prolonging the exposure time to 72 h and were equal to 62.8 μg/mL (95% confidence intervals, CI = 53.8–73.3 μg/mL) and 5.4 μg/ml (95% CI = 2.2–12.9 μg/mL) for FLG and GO3, respectively. The significant difference between the two EC_50_ values (*p* < 0.001) demonstrates that FLG is significantly less potent than GO3 in reducing mitochondrial activity, being twelve times less active, suggesting that the cytotoxic potential is dependent on the oxidation state of the material.

In panels D-F of Fig. 2, a comparison between similarly oxidized compounds differing by lateral dimensions (GO1, GO2 and GO3) is shown. GOs exert significant effects already after 24 h exposure, inducing a significant reduction of the mitochondrial activity, starting at concentrations of 3 μg/mL (21% and 25% reduction for GO1 and GO3, respectively) and 10 μg/mL (29% reduction for GO2). Also in this case the EC_50_ computation was allowed only after 72 h exposure and were equal to 12.9 μg/mL (95% CI = 7.1–23.4 μg/mL), 18.6 μg/mL (95% CI = 12.2–28.2 μg/mL) and 5.4 μg/ml (95% CI = 2.2–12.9 μg/mL) for GO1, GO2 and GO3, respectively. These values resulted significantly different only comparing GO2 vs GO3 (p < 0.05), suggesting only a partial role of lateral dimension on the cytotoxic potency. In addition, this significance could rise from the presence of large flakes (>2 μm) mainly for GO3 (Fig. 1).

Our observations are in line with previous studies demonstrating an association between the reduction of oxygen content and a reduced oxidative stress-dependent cytotoxicity of GOs on HUVEC endothelial cells^53^. On the contrary, Liao and coworkers (2011) suggested that a reduction of the oxidation state of GO could imply a stronger cytotoxicity on fibroblast^52^. This discrepancy, however, could be due to different synthetic procedures or to other properties, such as size and shape, as discussed by Das *et al*.^53^. Indeed, the graphene used by Liao and co-workers was obtained by acidic dehydration of GO, a material still containing many defects and a very high content of oxygen. This aspect, together with its irreversible aggregation observed in culture medium resulting in a significantly larger material (about 4 μm)^52^, impaired a direct comparison with our data. In line with this suggestion, GO3, the most active compound, is characterized by the largest average dimension, due to the presence of flakes larger than 2 μm, scarcely present in the other materials. This observation suggests that also the size could be a critical feature, even though further studies are needed to clarify this aspect. On the other hand, it seems that using carbon fibers as starting material, such as in the case of GO2, does not induce significant differences in the behaviour of the material.

### Effects of FLG and GOs on cell proliferation

**Figure 3:**
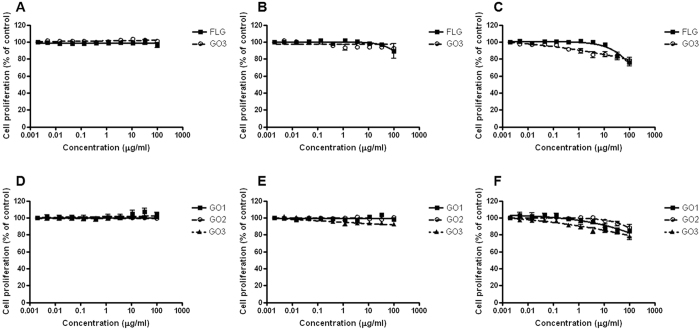
Effect of FLG and GOs on HaCaT cells proliferation evaluated by SRB incorporation assay. Comparison between the less (FLG) and most (GO3) oxidized GBMs after 24 h (**A**), 48 h (**B**) and 72 h (**C**) exposure. Comparison between similarly oxidized GBMs differing by average lateral dimension (GO1, GO2, GO3) after 24 h (**D**), 48 h (**E**) and 72 h (**F**) exposure. Data are the mean ± SE of 3 independent experiments performed in triplicate.

Intriguingly, the significant mitochondrial dysfunction induced by FLG and GOs does not appear to imply a consequent reduction of cell proliferation (SRB assay), which could be expected from a mitochondrial activity perturbation. In fact, after 24 and 48 h exposure, FLG and GOs did not induce significant effects on cell proliferation. Similarly, after 72 h no significant effects were observed up to 30 μg/mL, whereas only at the highest concentration (100 μg/mL) a slight reduction of cell proliferation was observed: 24, 15, 12 and 22% for FLG, GO1, GO2 and GO3, respectively (Fig. 3). These results are in agreement with previous studies showing very weak cytotoxic and anti-proliferative effects of graphene films^54^ and GO^55^ in murine NIH-3T3 fibroblasts or U87 and U118 glyoma cells, respectively.

### Effects of FLG and GOs on plasma membrane integrity

**Figure 4:**
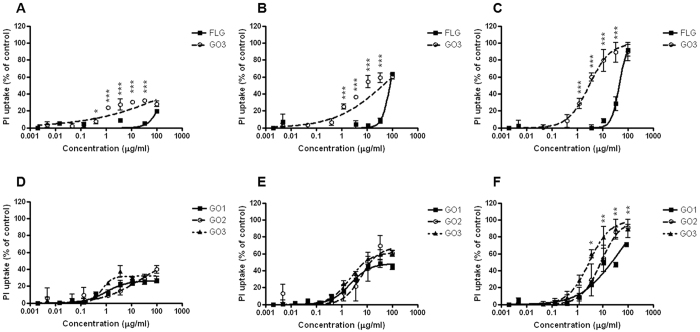
Effect of FLG and GOs on HaCaT cells plasma membrane integrity evaluated by PI uptake assay. Comparison between the less (FLG) and most (GO3) oxidized GBMs after 24 h (**A**), 48 h (**B**) and 72 h (**C**) exposure. Comparison between similarly oxidized GBMs differing by average lateral dimension (GO1, GO2, GO3) after 24 h (**D**), 48 h (**E**) and 72 h (**F**) exposure. Data are the mean ± SE of 3 independent experiments performed in triplicate. Statistical differences: *p < 0.05; **p < 0.01; ***p < 0.001 (Two-way ANOVA and Bonferroni’s post test).

In contrast with the absence of anti-proliferative properties, the effects of FLG and GOs on HaCaT cells seem to imply a significant damage at the plasma membrane levels, as evaluated by means of propidium iodide (PI) cell uptake (Fig. 4).

Figure 4 (panels A–C) shows a comparison between the less (FLG) and most oxidized (GO3) GBMs after 24 (panel A), 48 (panel B) and 72 hours of exposure (panel C). While 24 h exposure of HaCaT keratinocytes to FLG or GO3 induced a negligible PI uptake into the cells, a significant concentration-dependent effect was observed starting from 48 h exposure. In particular, after 48 h, FLG induced a significant effect, starting from the concentration of 30 μg/mL (8% of PI uptake) while GO3 induced a slight but significant PI uptake starting from the concentration of 1 μg/mL (25% PI uptake increase). However, also in this case, the low effect did not allow the exact evaluation of the EC_50_ values. By contrast, when increasing the exposure time up to 72 h, a slight increase of PI uptake was observed with EC_50_ values of 45.5 μg/mL (95% CI = 38.2–54.2 μg/mL) and 2.9 μg/mL (95% CI = 2.1–4.2 μg/mL) for FLG and GO3, respectively. These values, being significantly different (*p* < 0.001), confirm the findings of the WST-8 assay, demonstrating that GO3 and FLG are the most and the less toxic compounds, respectively, therefore confirming the hypothesis that GBMs toxic potential is dependent on their oxidation state.

In panels D-F of Fig. 4, a comparison between similarly oxidized compounds differing by lateral dimensions (GO1, GO2 and GO3) is shown. Again, the EC_50_ computation was allowed only after 72 h exposure and were equal to 23.5 μg/mL (95% CI = 15.8–34.9 μg/mL), 8.7 μg/mL (95% CI = 5.9–12.9 μg/mL) and 2.9 μg/mL (95% CI = 2.1–4.2 μg/mL) for GO1, GO2 and GO3, respectively. Even though significant differences were found comparing the EC_50_ values of GO1 vs GO2 (p < 0.01), GO1 vs GO3 (p < 0.001) and GO2 vs GO3 (p < 0.001), the little difference among these values suggests only a slight role of GBMs dimension, as already discussed for the WST-8 assay.

In addition, the EC_50_ values estimated for the PI uptake assay are comparable to those calculated for the WST-8 assay. This observation leads to the hypothesis that, at least after 72 h exposure, FLG and GOs could interfere with the plasma membrane leading to a damage that ultimately impairs mitochondrial activity, without a consequent influence on cell proliferation. To the best of our knowledge, this is the first study demonstrating a significant plasma membrane damage, even though previous studies reported negligible effects of graphene and GOs on other cell models^50^,^56^,^57^,^58^,^59^. However, these findings were obtained using other methods (i.e., lactate dehydrogenase cell release), which could be possibly less sensitive than PI uptake measurement.

**Figure 5:**
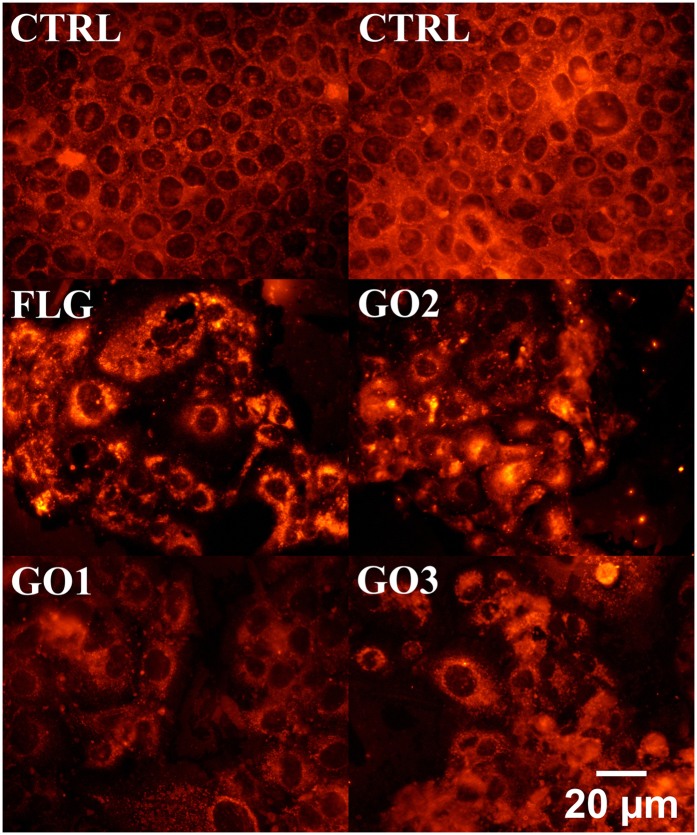
Epifluorescence micrographs of HaCaT cells exposed to 10 μg/mL of FLG, GO1, GO2 or GO3 for 72 h. Plasma membrane of HaCaT cells is labeled with the fluorescence dye DiL. Original magnification: 60x. Scale bar: 20 μm.

To further characterize the plasma membrane damage in HaCaT cells exposed to FLG and GOs, morphological analyses were carried out by epifluorescence microscopy after probing plasma membranes of HaCaT cells with DiL fluorescence dye. As shown in Fig. 5, 10 μg/mL FLG, GO1, GO2 or GO3 exposure for 72 h impaired the HaCaT cell membrane integrity and morphology. In particular, treated cells lost their typical flattened and cuboidal form, becoming swollen and presenting nuclear perturbations, characterized by an irregular shape.

### Interaction of FLG and GOs with cell membrane

**Figure 6:**
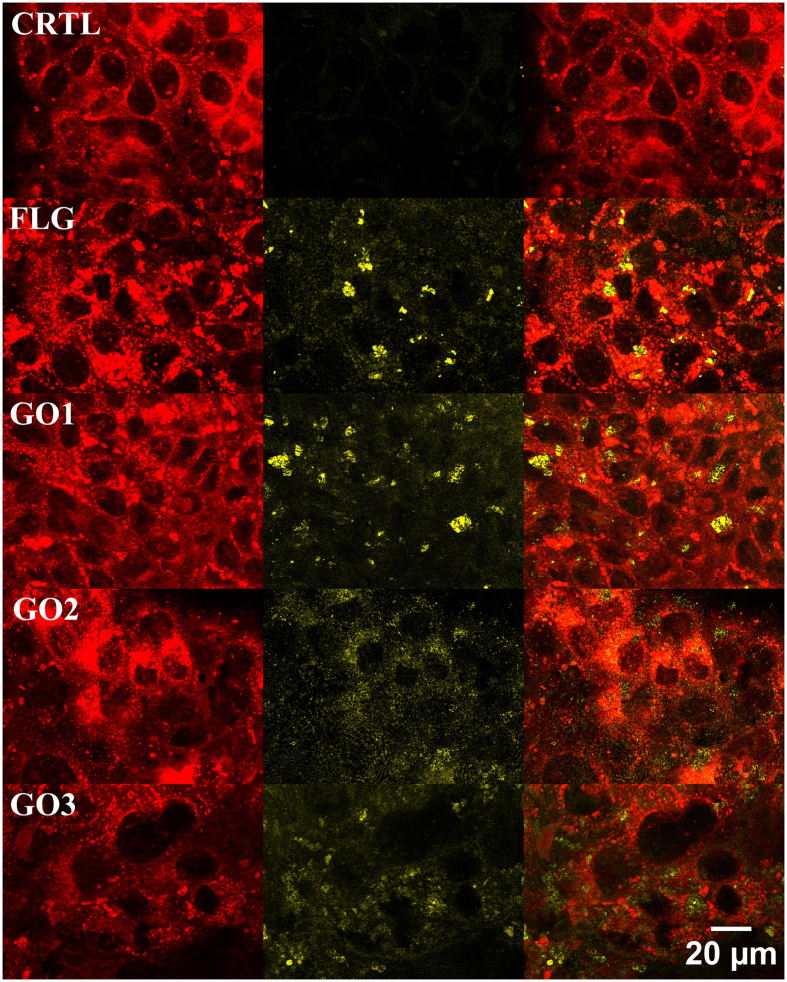
Reconstructed confocal micrographs of HaCaT cells exposed to 10 μg/mL FLG, GO1, GO2 or GO3 for 72 h. Plasma membrane of HaCaT cells is labeled with the fluorescence dye DiL (red, left panel); GBMs are visualized by reflection mode acquisition (yellow, middle panel); confocal reconstruction of red DiL labeled HaCaT cells merged with yellow reflecting GBMs (merged Figures, right panel). Original magnification: 60x. Scale bar: 20 μm.

To investigate the interactions between GBMs and the plasma membrane, HaCaT cells exposed to FLG or GOs (10 μg/mL) for 72 h were subjected to confocal microscopy analysis, in which cells were labelled with the membrane fluorescent DiL dye and GBMs were visualised in yellow exploiting their reflection mode during the confocal acquisition. As shown in Fig. 6, both FLG and GOs were able to interact with cells: by merging the red fluorescence given by the plasma membranes and the reflected yellow light by GBMs, FLG and GOs appeared diffusely associated with the cells membrane. Moreover, considering the repeated washings required during the fixation procedure for the confocal analysis, FLG and GOs appear to be strongly attached to the cells. This observation is supported also by a recent computational molecular dynamics simulation as well as electron microscopy imaging, showing the ability of few-layer graphene microsheets to interact and penetrate the plasma membrane of different cell types^60^. Similarly, transmission electron microscopy analysis showed that GO and graphene nanoplatelets penetrate through the membrane into the cytosol of human hepatocellular carcinoma HepG2 cells^61^. In line with these observations, our confocal images, representing a slice along the *z* stack internal to the cells, show that FLG and GOs can be found also inside the cells, suggesting a possible penetration inside skin keratinocytes.

### Long-term cytotoxicity of FLG and GOs

**Figure 7:**
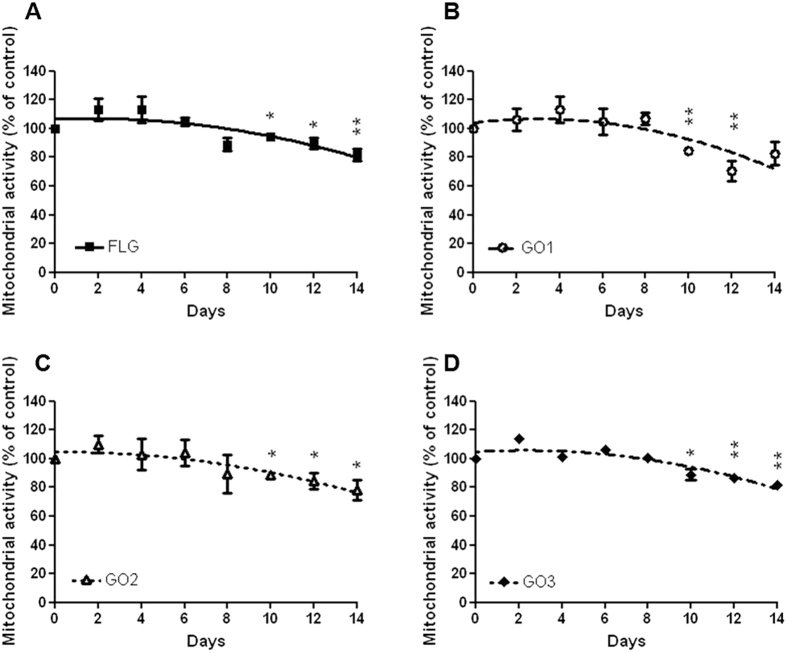
Long-term effect of FLG and GOs on HaCaT mitochondrial activity evaluated by the WST-8 assay. Cells were exposed for increasing time intervals up to 14 days to 0.1 μg/mL FLG (**A**), GO1 (**B**), GO2 (**C**) and GO3 (**D**). Data are the mean ± SE of 3 independent experiments performed in triplicate. Statistical differences: *p < 0.05; **p < 0.01 (One-way ANOVA and Bonferroni’s post test).

After acute cell exposure to FLG and GOs, significant cytotoxic effects were observed only after long exposure times (72 hours) to high GBMs concentrations. To investigate if long-term exposures to low GBMs concentrations could affect cell viability, HaCaT cells were exposed to the highest concentration not giving significant effects (0.1 μg/mL) for increasing exposure times up to 14 days. Figure 7 shows the effects of FLG, GO1, GO2 and GO3 by means of mitochondrial activity, evaluated by the WST-8 assay. All the materials induced only slight reductions of mitochondrial activity, being significant only after 10 days exposure (mitochondrial activity reduction of 6%, 16%, 12% and 12% for FLG, GO1, GO2 and GO3, respectively). These data suggest that long-term exposure to low GBMs concentration induces only slight cytotoxic effects.

## Conclusion

In conclusion, this study provides the first comparative data of different GBMs effects on a skin *in vitro* model. Even though it is very difficult to predict a possible human exposure since no industrial-scale adoption of graphene has taken place, so far^62^, these results demonstrate significant cellular damage induced by FLG and GOs only at high concentrations (>30 μg/mL and >1 μg/mL for FLG and GOs, respectively) on skin keratinocytes after an exposure time as long as 72 h, with variable potencies depending on GBMs oxidation state. This cytotoxicity seems to be lower than other carbon nanomaterials at the skin level. For instance, it has been reported that single-wall carbon nanotubes (SWCNTs) significantly reduce viability of HEK keratinocytes at ng/mL concentrations already after 24 h^63^, whereas the GBMs tested in this study displayed weak cytotoxic effects at μg/mL concentrations. Similarly, multi-wall carbon nanotubes (MWCNTs) reduce viability of IHK^64^ and HEK keratinocytes after 24 h, with significant cellular internalization already after 1 h exposure^65^ and oxidative stress being significant after only 4 h exposure^66^. All together, these observations lead to conclude that, also compared to other carbon nanomaterials, GOs and especially FLG exert very weak cytotoxicity on skin keratinocytes. Even though GBMs cytotoxicity could be reduced by a protein corona due to the presence of serum inside culture medium^67^,^68^, the present results suggest an acceptable biocompatibility of these materials, both after short acute exposure times (i.e. 24 h) and after long-term exposure to low GBMs concentrations (0.1 μg/mL, up to 10 days). In addition, these results were obtained on proliferative keratinocytes, a simplified *in vitro* model excellent to study mechanism of toxicity at the skin level, but in which the lack of barrier properties could increase FLG and GOs toxicity. Experiments are in progress to further characterize their effects at the cutaneous level.

## Materials and Methods

### Chemicals

FLG and GO1 were prepared starting from graphite (from Bay Carbon, Inc. SP-1 graphite powder batch N°04100, lot N°011705, www.baycarbon.com). FLG was prepared by ball-milling treatment of graphite through interaction with melamine (purchased by Sigma-Aldrich and used as received without further purification) in solvent free conditions^42^. GO1 was prepared by a modified Hummers’ method^12^. GO2 and GO3 were obtained from Antolin group (Burgos, Spain, www.grupoantolin.com) and Graphenea group (San Sebastián, Spain, www.graphenea.com), respectively.

All reagents of analytical grade for *in vitro* experiments were purchased from Sigma-Aldrich (Milan, Italy), if not otherwise specified.

### Synthesis of FLG and GO

#### FLG

The graphene used in this study was obtained by a methodology^42^ that uses mechanochemical activation by ball-milling to exfoliate graphite through interactions with melamine (2,4,6-triamine-1,3,5-triazine). In a typical experiment, 7.5 mg of graphite and 0.16 mmol of melamine were ball-milled in a Retch PM100 Planetary Mill (Haan, Germany) at 100 rpm for 30 minutes in air atmosphere. The resulting solid mixtures were dispersed in 20 mL of Milli-Q-water to produce stable black suspensions. The as-prepared dispersions can be filtered and washed in hot water to remove melamine. Graphene water dispersions are obtained with a final concentration of 0.09 mg/mL in Milli-Q-water.

#### GO1

It was prepared using the improved Hummer’s method^12^. A mixture of concentrated H_2_SO_4_/H_3_PO_4_ (180:20 mL), was added into a mixture of powdered graphite (1.5 g) and KMnO_4_ (1.8 g). Then, the resulting mixture was heated to 50 °C and stirred for 12 h. The reaction was then cooled to RT and poured in ice water (200 mL) with addition of H_2_O_2_ (0.5 mL, 30%). The mixture was filtered and washed with water. The resulting wet solid was re-dissolved in water (200 mL) and dialyzed until neutral pH and colorless aqueous solution was observed. The dialyzed suspension was centrifuged (4000 rpm, 1 h) in order to separate the graphite material. The supernatant was filtered and washed with ethyl ether, obtaining 2.6 g of brown solid.

### Elemental analysis

Elemental Analysis was performed with a LECO CHNS-932 analyzer (Model No: 601-800-500).

### Thermogravimetric analysis (TGA)

The thermogravimetric analyses were performed with a TGA Q50 (TA Instruments, New Castle, USA) at 10 °C/min under nitrogen flow, from 100 °C to 800 °C.

### X-Ray photoelectron spectroscopy

#### For FLG

X-Ray photoelectron spectra (XPS) were obtained with a VG Escalab 200 R spectrometer equipped with a hemispherical electron analyzer with a pass energy of 50 eV and a Mg K α (h ν = 1254.6 eV) X-ray source, powered at 120 W. High-resolution spectra envelopes were obtained by curve fitting synthetic peak components using the software *XPS peak*. Symmetric Gaussian–Lorentzian curves were used to approximate the line shapes of the fitting components. Atomic ratios were computed from experimental intensity ratios and normalized by atomic sensitivity factors.

#### For GO1, GO2 and GO3

XPS experiments were performed in a SPECS Sage HR 100 spectrometer with a non-monochromatic X-ray source of Magnesium with a Kα line of 1253.6 eV energy and 250 W. The samples were placed perpendicular to the analyzer axis and calibrated using the 3d5/2 line of Ag with a full width at half maximum (FWHM) of 1.1 eV. An electron flood gun was used to compensate for charging during XPS data acquisition. The selected resolution was 30 and 15 eV of Pass Energy and 0.5 and 0.15 eV/step for the survey and high resolution spectra, respectively. Measurements were made in an ultra high vacuum (UHV) chamber at a pressure below 8·10-8 mbar. Fitting of the XPS data were done using *CasaXPS 2.3.16 PR 1.6 software*. For our data, the Shirley-type background subtraction was used and all curves were defined as 40% Lorentzian, 60% Gaussian. Atomic ratios were computed from experimental intensity ratios and normalized by atomic sensitivity factors.

### Raman Spectroscopy

#### For FLG and GO2

Raman spectra were recorded for graphene samples on silicon surface (Si-Mat silicon wafers, CZ) by drop-casting after complete evaporation of the water. Measurements were carried out using a 100x objective at 532 nm laser excitation using a SENTERRA Raman Microscope.

#### For GO1 and GO3

Raman spectroscopic measurements were acquired on a LabRAM HR Raman spectrometer (Horiba Jobin–Yvon) with laser excitation energy of 2.33 eV (l = 532 nm, ArKr laser, coherent). A 100x objective lens was used with a laser spot of about 1 μm. The laser power was 1 mW and the spectral resolution was 1 cm^−1^. Each sample was deposited as powder on a glass slide and was measured in multiple regions.

### Transmission electron microscopy (TEM)

For TEM analyses, cell culture media (Dulbecco’s Modified Eagle Medium, DMEM) dispersions of graphene were diluted as necessary and dip-cast on a Lacey copper grid (3.00 mm, 200 mesh, coated with carbon film), and dried under vacuum. Sample were investigated by High-Resolution Transmission Electron Microscopy (HRTEM) JEOL 2100. Lateral dimension distribution was carried out using Fiji-win32.

### Cell Culture

The human skin HaCaT cell line was purchased from Cell Line Service (DKFZ, Eppelheim, Germany) and all cell culture reagents were from Euroclone (Milan, Italy). Cells were maintained in DMEM high glucose supplemented with 10% FBS, 2 mM L-glutamine, 100 IU/mL penicillin and 0.1 mg/mL streptomycin. Cell cultures were maintained according to standard procedures in a humidified incubator at 37 °C with 5% CO_2_, performing cell passages once a week. If not otherwise specified, for cytotoxicity experiments, cells were seeded in 96-wells plates at a density of 5 × 10^3^ cells/well. Experiments were carried out between passages 50 and 65.

### Cells exposure to FLG and GOs

For cytotoxicity assays, cells were exposed to FLG (0.005 to 90 μg/mL) or GOs (0.005 to 100 μg/mL) up to 72 h, if not otherwise specified.

### WST-8 reduction assay

FLG and GOs effect on mitochondrial activity of HaCaT cells was evaluated by the 2-(2-methoxy-4-nitrophenyl)-3-(4-nitrophenyl)-5-(2,4-disulfophenyl)-2H-tetrazolium (WST-8) reduction assay. After exposure to FLG or GOs, cells were washed three times with PBS (200 μL/well) and incubated for 4 h with fresh medium (100 μL/well) containing 10 μL of WST-8 reagent. Absorbance was subsequently read at 450 nm by an Automated Microplate Reader EL 311 s (Bio-Tek Instruments, Winooski, VT, USA). Data are reported as % of mitochondrial activity in cells exposed to FLG or GOs with respect to untreated control cells.

### Sulforhodamine B (SRB) assay

FLG and GOs effects on HaCaT cells proliferation were evaluated by the sulforhodamine B assay, as previously described^69^,^70^. Briefly, after exposure to FLG or GOs, cells were washed three times with PBS (200 μL/well), fixed with 50% (v/v) trichloroacetic acid for 1 h at 4 °C and stained for 30 min with 0.4% SRB in 1% (v/v) acetic acid. After washings with 1% (v/v) acetic acid, the protein-bound dye was dissolved in 10 mM TRIZMA base solution and the absorbance was read by an Automated Microplate Reader EL 311 s (Bio-Tek Instruments, Winooski, VT, USA) at 570 nm. Data are reported as % of cell proliferation after FLG or GOs exposure with respect to untreated control cells.

### Propidium iodide (PI) uptake

Cell membrane damages were evaluated by measuring PI fluorescence inside the cells, as previously described^70^,^71^. Briefly, after exposure to FLG or GOs, cells were washed three times with PBS and then exposed to 3.0 × 10^−6^ M PI in PBS for 30 min at 37 °C. As a positive control, 0.1% (vol/vol) Triton-X in PBS were added. Fluorescence intensity was read by a Fluorocount Microplate Fluorometer (Packard, Germany) with excitation wavelength of 485 nm and emission wavelength of 590 nm. Each sample was subsequently permeabilized with 0.1% Triton-X for 30 min to measure total fluorescence (index of total cell content). Data are reported as % of PI with respect to positive control cells, after normalization on cell content.

### Epifluorescence microscopy analysis

Cells (5 × 10^4^ cells/well) were seeded for 24 h in 24-wells plates. After staining of plasma-membrane with 1 μM 1,1′-Dioctadecyl-3,3,3′,3′-tetramethylindocarbocyanine perchlorate (DiL), cells were exposed to FLG or GOs (10 μg/mL) for 72 h. Cells were then washed three times with PBS (1 mL/well), fixed for 30 min at RT in 4% paraformaldehyde (PFA) and washed twice with PBS (1 mL/well). Samples were mounted in mowiol on coverslips of 1 mm thickness. Cell membrane morphology was observed by an epifluorescent microscope (Eclipse E800, Nikon) at 60x magnification.

### Confocal microscopy analysis

Cells (5 × 10^4^ cells/well) were seeded for 24 h in 24-wells plates, exposed to FLG or GOs (10 μg/mL) for 72 h after probing cell membranes with 1 μM DiL as described above. Images were taken by a confocal microscope (Eclipse C1si, on an inverted microscope TE2000U, Nikon) at 60x magnification. FLG and GOs were visualized by the reflection mode property during the confocal acquisitions. Reconstructions of the images were performed offline using the image-processing package Fiji.

### Statistical analysis

Results are presented as mean ± SE from at least three independent experiments performed in triplicate. Non-linear regression of concentration-effect data was performed using GraphPad Prism version 4.00 for computing the concentration giving the 50% of the effect (EC_50_). Statistical differences among EC_50_ values were evaluated by Student *t*-test (significant differences, p < 0.05), data obtained by comparison of different GBMs were analyzed by a two-way ANOVA analysis followed by Bonferroni’s post-test (PrismGraphPad, Inc.; San Diego, CA, USA) while data obtained by long-term analysis were analyzed by a one-way ANOVA analysis followed by Bonferroni’s post-test (PrismGraphPad, Inc.; San Diego, CA, USA) and significant differences were considered at p < 0.05.

## Additional Information

**How to cite this article**: Pelin, M. *et al*. Differential cytotoxic effects of graphene and graphene oxide on skin keratinocytes. *Sci. Rep.* **7**, 40572; doi: 10.1038/srep40572 (2017).

**Publisher's note:** Springer Nature remains neutral with regard to jurisdictional claims in published maps and institutional affiliations.

## Supplementary Material

Supplementary Materials

## Figures and Tables

**Table 1:** Summarized materials properties of GBMs.

GBM	Elemental Analysis ± SD (wt%)					Atomic ratio (at. %)^a^			TGA weight lost (%)^b^	Lateral Dimension ± SD (nm)^c^
	C	H	N	O	S	N/C	O/C	S/C		
FLG	94.03 ± 0.64	0.32 ± 0.03	0.31 ± 0.03	5.34 ± 0.64	—	0.011	0.074	—	7	552 ± 245
GO1	43.20 ± 0.07	3.67 ± 0.07	0.07 ± 0.01	42.70 ± 0.02	10.37 ± 0.03	—	0.53	0.047	50	622 ± 581
GO2	47.71 ± 0.03	3.04 ± 0.02	0.15	48.84 ± 0.02	0.27 ± 0.03	—	0.51	—	52	845 ± 427
GO3	41.88 ± 1.06	3.04 ± 0.14	0.04	52.23 ± 0.46	2.82 ± 0.52	—	0.51	0.030	45	979 ± 498

^a^Ratios determined from the XPS survey spectra.

^b^Values determined at 700 °C.

^c^Values determined by HRTEM on 150 sheets.
