# Supplementary material for: Differential cytotoxic effects of graphene and graphene oxide on skin keratinocytes
Source: Sci Rep. 2017 Jan 12;7:40572. doi: 10.1038/srep40572 (PMC5227695; doi:10.1038/srep40572)

**Differential cytotoxic effects of graphene and graphene oxide on skin keratinocytes**

Marco Pelin,^1,2^ Laura Fusco,^2^ Veronica León,^3^ Cristina Martín,^3^ Alejandro Criado,^3,4^ Silvio Sosa,^1^ Ester Vázquez,*^,3^ Aurelia Tubaro,*^,1^ Maurizio Prato*^,2,4,5^

^1^ Department of Life Sciences, University of Trieste, 34127 Trieste, Italy

^2^ Department of Chemical and Pharmaceutical Sciences, University of Trieste, 34127 Trieste, Italy

^3^ Department of Organic Chemistry, Facultad de Ciencias y Tecnologías Químicas-IRICA. University of Castilla-La Mancha, 13071 Ciudad Real, Spain

^4^ CIC BiomaGUNE, Parque Tecnológico de San Sebastián, Paseo Miramón, 182, 20009 San Sebastián (Guipúzcoa), Spain

^5^ Basque Foundation for Science, Ikerbasque, Bilbao 48013, Spain

**Corresponding authors:** Prof. Ester Vazquez, email: ester.vazquez@uclm.es; Prof. Aurelia Tubaro, email: tubaro@units.it; Prof. Maurizio Prato, email: prato@units.it

**Supporting Information**

**Table 1S.** Atomic ratios determined from the XPS survey spectra for G, GO(1), GO(2) and GO(3).

| **GBN** | **O/C** | **S/C** | **N/C** |
| --- | --- | --- | --- |
| G | 0.074 | - | 0.011 |
| GO1 | 0.53 | 0.047 | - |
| GO2 | 0.51 | - | - |
| GO3 | 0.51 | 0.030 | - |

**Figure S1.** TGA plot of GBNs (A). Representative Raman spectra of GBNs where spectra for GO(1), GO(2) and GO(3) were performed with a baseline correction (B).

**
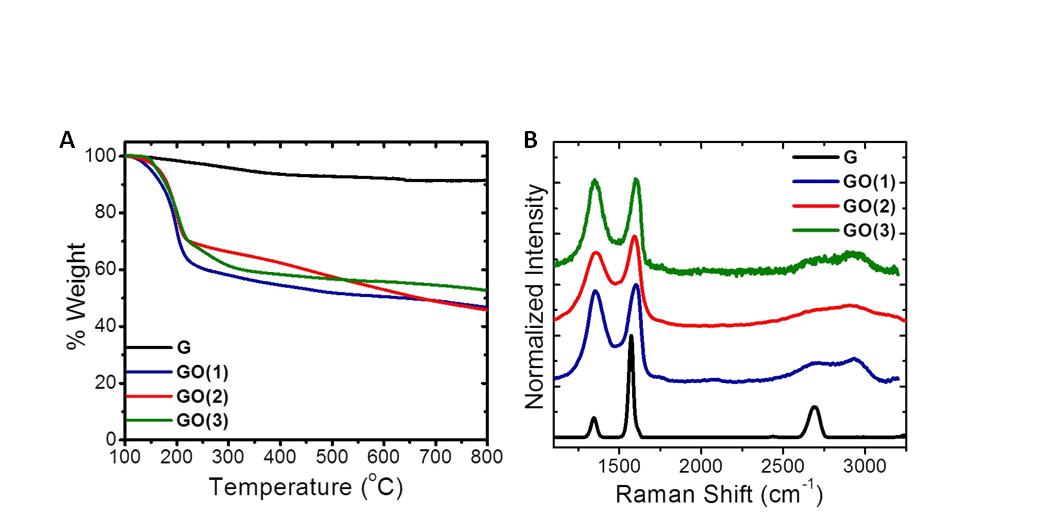
**

**Figure S2.** XPS survey spectra (A) and C1s high resolution spectra (B) for G, GO(1), GO(2) and GO(3). O1s high resolution spectra for GO(1), GO(2) and GO(3) (C). S2p high resolution spectra for GO(1) and GO(3) (D). N1s high resolution spectra for G (E).


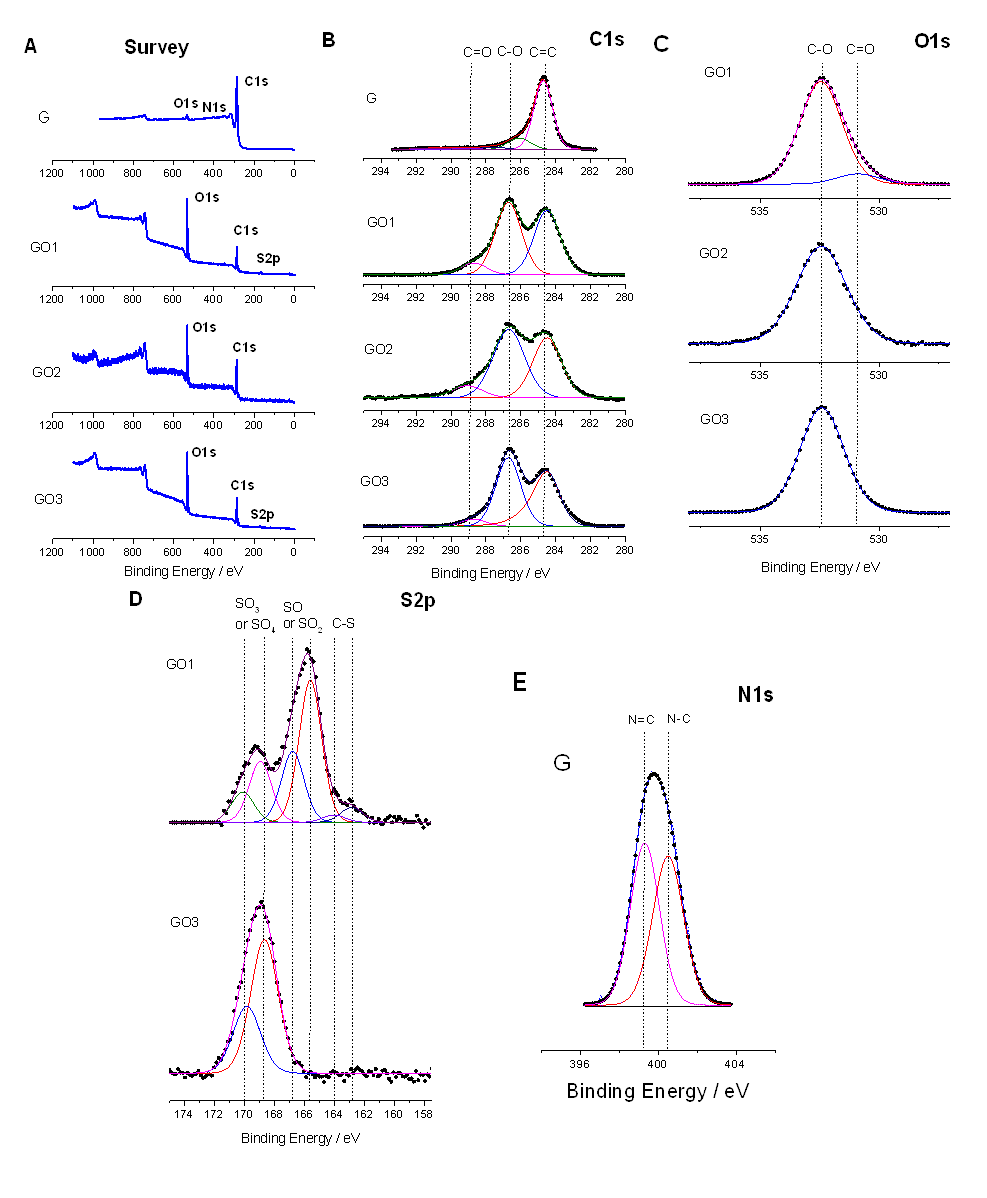

Supplement: Supplementary Materials [file srep40572-s1.doc]
